# Advanced hepatocellular carcinoma with response to lenvatinib after atezolizumab plus bevacizumab

**DOI:** 10.1097/MD.0000000000027576

**Published:** 2021-10-22

**Authors:** Shigeki Yano, Tomokazu Kawaoka, Yusuke Johira, Ryoichi Miura, Masanari Kosaka, Yuki Shirane, Serami Murakami, Kei Amioka, Kensuke Naruto, Yuwa Ando, Yumi Kosaka, Kenji Yamaoka, Kenichiro Kodama, Shinsuke Uchikawa, Hatsue Fujino, Atsushi Ohno, Takashi Nakahara, Eisuke Murakami, Wataru Okamoto, Masami Yamauchi, Michio Imamura, Keiichi Mori, Kouji Arihiro, Shintaro Kuroda, Tsuyoshi Kobayashi, Hideki Ohdan, Hiroshi Aikata

**Affiliations:** a Department of Gastroenterology and Metabolism, Hiroshima University, Japan; b Anatomical Pathology, Hiroshima University, Japan; c Gastroenterological and Transplant Surgery, Hiroshima University, Japan.

**Keywords:** adrenal metastasis, atezolizumab plus bevacizumab, conversion surgery, hepatocellular carcinoma, lenvatinib

## Abstract

**Rationale:**

Various treatments are available for treating hepatocellular carcinoma (HCC). The immune checkpoint inhibitor combination of atezolizumab plus bevacizumab was recently approved for the treatment of unresectable HCC, but there are few reports on the failure of the combination treatment. Here, we present a case of unresectable HCC with adrenal metastasis that was eventually operated on after lenvatinib (LEN) treatment that followed failed treatment with atezolizumab plus bevacizumab.

**Patient concerns:**

A 68-year-old man was diagnosed with non-alcoholic steatohepatitis-based HCC with adrenal metastasis.

**Diagnosis:**

Cirrhosis was classified as Child-Pugh score of 5. HCC was diagnosed as Barcelona Clinic Liver Cancer stage C.

**Interventions:**

We initiated treatment with atezolizumab plus bevacizumab. Liver dysfunction appeared 2 days after the first administration but was improved by intravenous rehydration and did not appear after the second course. The HCC shrank, but the adrenal metastasis grew bigger after the fourth course, so we changed the therapy to LEN. After HCC and adrenal metastasis were necrotic by LEN, conversion surgery was performed.

**Outcomes:**

After successful conversion therapy, the general condition of the patient was good, and has been carefully followed for 4 months to date without any evidence of further recurrences.

**Lessons:**

This case showed that even if atezolizumab plus bevacizumab is not effective, multidisciplinary treatment such as LEN and conversion surgery is possible. Given the efficacy of LEN after atezolizumab plus bevacizumab, it is important to consider that there is a possibility of cure even when first-line treatment is not effective for a patient with unresectable HCC.

## 1 Introduction

Although its mortality has recently become more stable, hepatocellular carcinoma (HCC) remains a leading cause of cancer-related deaths worldwide.^[1]^ Currently, there are numerous treatments for HCC, including surgical resection, liver transplantation, radiofrequency ablation, percutaneous ethanol injection, transcatheter arterial chemoembolization, radiation therapy, hepatic arterial infusion chemotherapy, and molecular targeted agent. Although early-stage disease may be curable, most patients present with unresectable disease and have a poor prognosis. No effective therapy existed for patients with advanced-stage HCC until May 2009, when the tyrosine kinase inhibitor sorafenib (SOR) was approved as a systemic treatment for unresectable HCC, and lenvatinib (LEN) was found to be non-inferior to SOR as a treatment for unresectable HCC in a phase III trial (REFLECT).^[2]^

Recently, immune checkpoint inhibitors (ICIs) have achieved promising outcomes. The combination of atezolizumab, an antibody against programmed death-ligand 1 (PD-L1), with bevacizumab, an antibody against vascular endothelial growth factor, has become the standard of care as a first-line therapy for unresectable HCC. Atezolizumab plus bevacizumab is associated with better progression-free and overall survival (OS) outcomes, response rate, and preservation of quality of life than SOR (IMbrave150).^[3]^ However, the confirmed objective response rates were only 27.3% (95% CI, 22.5–32.5) according to the independent assessment with Response Evaluation Criteria in Solid Tumors, version 1.1 and 33.2% (95% CI, 28.1–38.6) according to the HCC-specific modified Response Evaluation Criteria in Solid Tumors (mRECIST),^[3]^ and some patients do not respond to atezolizumab plus bevacizumab. In this report, we describe a patient with unresectable HCC who successfully achieved conversion surgery after LEN treatment that followed the failure of atezolizumab plus bevacizumab treatment.

## 2 Case report

Written informed consent was obtained from the patient for publication of this case report and any accompanying images. The patient was a 68-year-old man with a medical history of type 2 diabetes and hypertension. In 20XX, he visited our hospital for the examination of liver dysfunction. Percutaneous liver biopsy led to the diagnosis of non-alcoholic steatohepatitis. Subsequently, he continued to see his family doctor. In 20XX+6, a medical examination revealed thrombocytopenia, and computed tomography (CT) showed a large liver tumor. Subsequently, he was referred to our hospital for further diagnosis and management.

A blood test at the first visit showed an elevated alanine aminotransferase level of 62 U/L and aspartate aminotransferase level of 64 IU/L. A total bilirubin level of 0.9 mg/dL, albumin level of 4.0 g/dL, and a prothrombin time level of 110% were normal. The serum level of alpha-fetoprotein (AFP) was 41.2 ng/mL, and that of des-gamma carboxyprothrombin (DCP) was 13,716 mAU/mL. He had a Child-Pugh score of 5, Child-Pugh grade A, and no hepatic encephalopathy or ascites (Table 1).

**Table 1:** Laboratory examinations.

**Complete blood cell count**			γ-GTP	237	IU/L	**Viral markers**	
WBC	5280	/μL	Na	139	mEq/L	HBs antigen	(−)
RBC	5.32 × 10^4^	/μL	K	4.1	mEq/L	HBs antibody	(−)
Hb	17.8	g/dL	Cl	105	mEq/L	IgGHBc antibody	(+)
Ht	51.7	%	TP	7.4	g/dL	HCV antibody	(−)
Plt	121 × 10^3^	/μL	Alb	4.0	g/dL	**Liver function**	
**Blood coagulation test**			BUN	15.5	mg/dL	ICG-R	27.7%
PT	110	%	Cr	0.88	mg/dL	Child-Pugh score	5
PT-INR	0.95		CRP	0.11	mg/dL	Child-Pugh grade	A
**Blood chemistry**			NH3	33	μmol/L	ALBI score	−2.62
T-Bil	0.9	mg/dL	HbA1c	6.7	%	ALBI grade	1
AST	64	IU/L	**Tumor markers**				
ALT	62	IU/L	AFP	41.2	ng/mL		
LDH	272	IU/L	AFP-L3	18.6	%		
ALP	348	IU/L	DCP	13716	mAU/mL		

**Figure 1:**
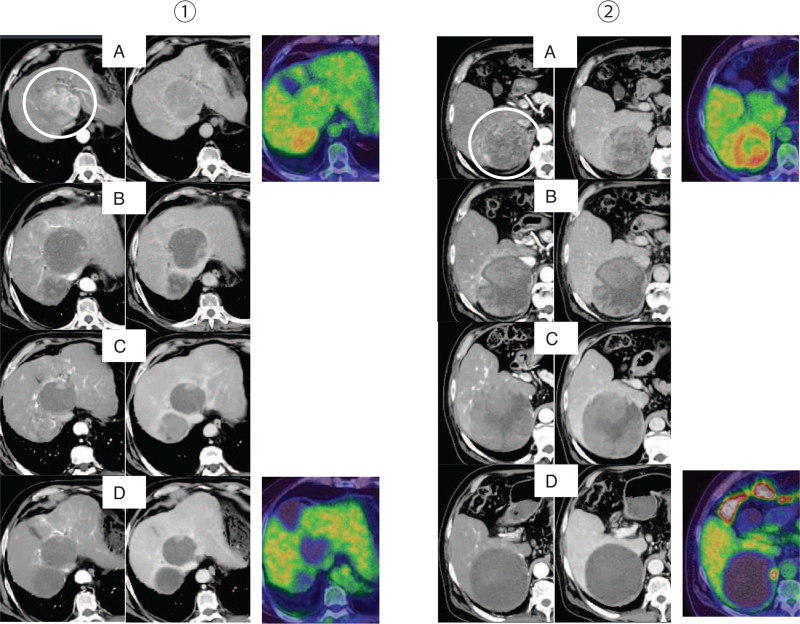
CT (early phase, delayed phase) and PET. (1) Intrahepatic HCC (S1) (2) Right adrenal metastasis. (A) CT and PET images at the initial diagnosis. (B) Six days after the first administration of atezolizumab plus bevacizumab. The internal contrast effects were diminished in both the intrahepatic HCC and the adrenal metastasis. (C) Evaluation of the efficacy after the fourth administration of atezolizumab plus bevacizumab. The intrahepatic HCC shrank while the adrenal metastasis grew larger than before, and the internal contrast effect was still present. (D) Evaluation of the efficacy of LEN after 1 month. The intrahepatic HCC maintained shrinkage, while the adrenal metastasis lost the internal contrast effect, and PET showed only light FDG uptake at the edge. CT = computed tomography, FDG = fluorodeoxyglucose, HCC = hepatocellular carcinoma, LEN = lenvatinib, PET = positron emission tomography.

CT showed a 72 mm liver tumor in S1 and an 83 mm right adrenal tumor, and both were internally enhanced (Fig. 1). Positron emission tomography showed fluorodeoxyglucose uptake in the adrenal metastasis (standardized uptake value maximum 4.5) but was negative for the intrahepatic HCC.

Percutaneous liver biopsy prior to treatment revealed a moderately differentiated HCC. We did not perform a biopsy of the adrenal tumor due to the fear of bleeding and blood pressure fluctuations.

**Figure 2:**
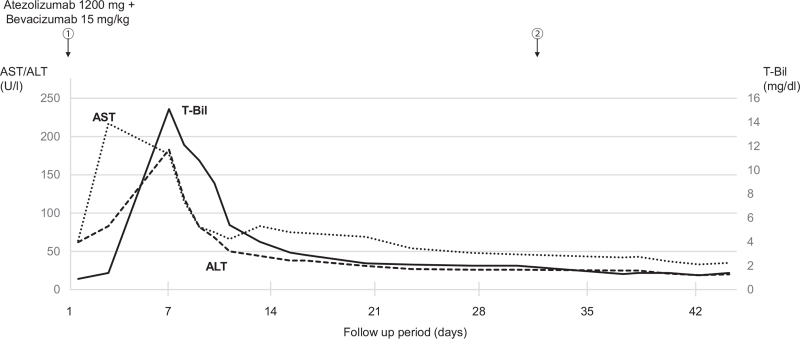
Clinical course after day 2 of the first administration of atezolizumab plus bevacizumab. The circled numbers are administration courses. Liver dysfunction peaked at 6 days of onset but declined rapidly thereafter. No liver dysfunction occurred after the second administration.

**Figure 3:**
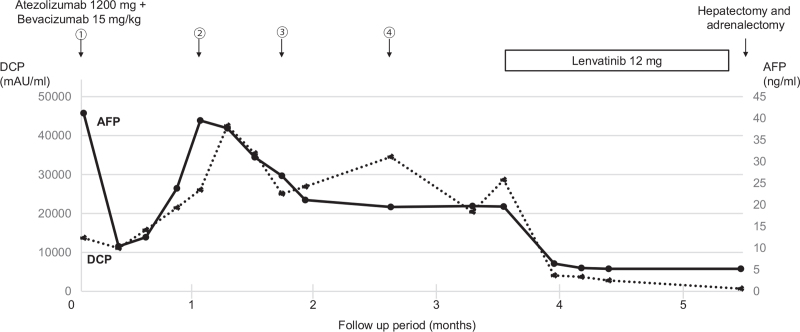
Timeline of the chemotherapy and changes in levels of DCP and AFP. The circled numbers are administration courses. AFP = alpha-fetoprotein, DCP = des-gamma carboxyprothrombin.

The patient was diagnosed with HCC with adrenal metastasis in Barcelona Clinic Liver Cancer stage C, and according to the fourth edition in 2020, which includes the 2017 edition of the Japan Society of Hepatology Guideline, atezolizumab (1200 mg) plus bevacizumab (15 mg/kg) were administered. Two days after the first administration, he developed right hypochondrial pain and fever. He visited our hospital, and a blood test showed severe liver dysfunction (Fig. 2). Potassium and uric acid levels were normal. Although immune-related adverse event or tumor lysis syndrome (TLS) were suspected, he got better with conservative treatment including rest and intravenous rehydration. No liver dysfunction was observed after the second administration. Thereafter, atezolizumab plus bevacizumab were administered as 1 course of 3 weeks without any adverse effects. After the fifth course, the intrahepatic HCC had shrunk, but the adrenal metastasis had grown larger than before, so we immediately changed from atezolizumab plus bevacizumab to LEN (Fig. 3). LEN was orally administered at 12 mg/day. There were grade 2 anorexia, hand-foot syndrome, and hypertension as adverse effects. After 1 month, the tumor markers were decreased, and a partial response according to mRECIST was achieved, so conversion surgery was considered. Positron emission tomography also showed only a slight fluorodeoxyglucose uptake at the edge. After confirming the absence of other lesions on hepatic angiography, we performed hepatectomy and adrenalectomy.

**Figure 4:**
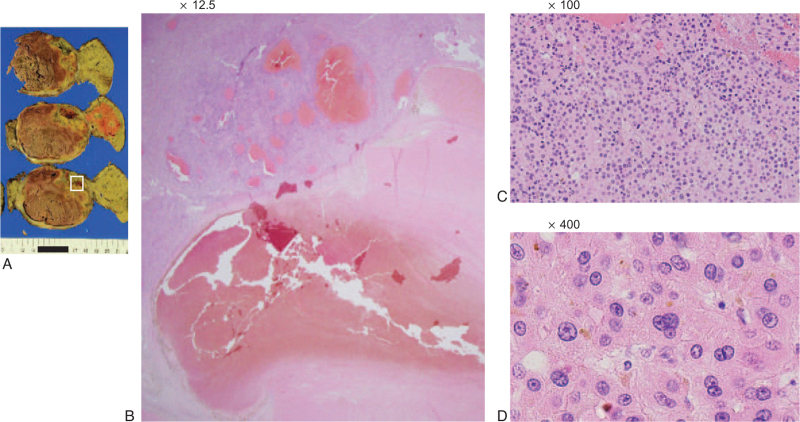
Histopathological findings of the intrahepatic HCC (S1). (A) Image of the resected specimen obtained after conversion surgery. (B) Hematoxylin and eosin (H&E) staining (×12.5), (C) H&E staining (×100), (D) H&E staining (×400). Intrahepatic HCC was of the moderately differentiated type and featured massive coagulative necrosis. HCC = hepatocellular carcinoma.

**Figure 5:**
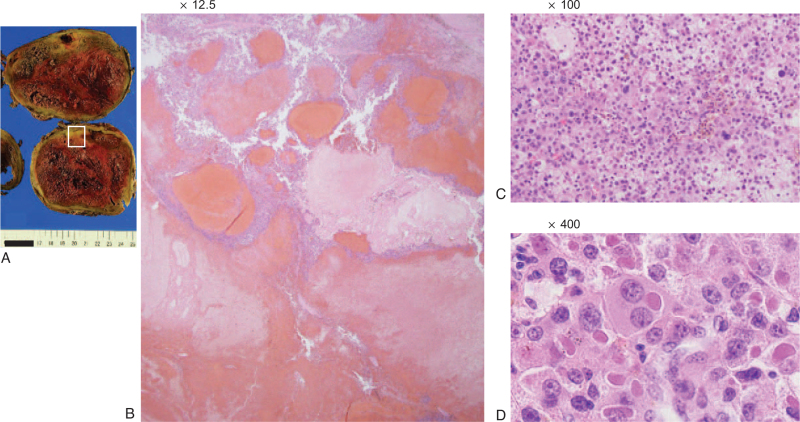
Histopathological findings of the right adrenal metastasis. (A) Image of the resected specimen obtained after conversion surgery. (B) Hematoxylin and eosin (H&E) staining (×12.5), (C) H&E staining (×100), (D) H&E staining (×400). There was little normal tissue and coagulative necrosis occupied a large area. The tumor cells were slightly more atypical than in the primary tumor.

Postoperative histopathological examination showed that the intrahepatic HCC was of the moderately differentiated type and featured massive coagulative necrosis (Fig. 4). In the right adrenal metastasis, there was little normal tissue, coagulative necrosis occupied a large area, and the tumor cells were slightly more atypical than in the primary tumor (Fig. 5).

During 4 months of postoperative follow-up without adjuvant therapy, the patient had no obvious complications. CT indicated that no recurrence and the serum level of alpha-fetoprotein and des-gamma carboxyprothrombin also remained within the normal limits.

## 3 Discussion

We describe a case of HCC with adrenal metastasis that showed a favorable response to systemic therapy and was able to undergo conversion surgery. To our knowledge, there are no other reports of HCC with adrenal metastasis that could be operated on in a patient treated with LEN after failure of atezolizumab plus bevacizumab.

In this case, liver dysfunction appeared 2 days after the first dose of atezolizumab plus bevacizumab, and immune-related adverse event or TLS was suspected. Although this case did not meet the diagnostic criteria for TLS,^[4]^ the presence of signs of tumor necrosis on CT and the fact that the patient improved without steroids suggests it is likely that the condition was similar to TLS. Such a reaction may occur after ICI administration; therefore, it is important to follow up carefully.

Atezolizumab plus bevacizumab, as first-line treatment, worked for the intrahepatic HCC but not for the adrenal metastasis. However, LEN worked for both as a second-line treatment. There has been a report on differences in the effects of ICIs in different organs,^[5]^ but so far, not all the facts are clear. On the other hand, the efficacy of LEN after atezolizumab plus bevacizumab has been reported.^[6]^ It is expected that anti-programmed death 1/programmed death 1 (PD-1) PD-L1 antibody remains bound to programmed death 1/PD-L1 on cancer cells continuously for a certain period,^[7]^ and this suggests that administration of LEN after atezolizumab plus bevacizumab may have synergistic effects similar to those of an ICI combined with a molecular targeted agent.^[8,9]^ As a multikinase inhibitor, LEN is more selective than bevacizumab for kinases important for angiogenesis, such as vascular endothelial growth factor receptor (VEGFR) and fibroblast growth factor receptor (FGFR).^[10]^ Furthermore, LEN improves the tumor microenvironment by itself.^[11]^ Therefore, the administration of LEN during the period of sustained effect after discontinuation of atezolizumab may have led to a synergistic effect with the improvement of tumor microenvironment. Aoki et al^[12]^ found that LEN displayed considerable antitumor effects with acceptable safety in patients with progressive and unresectable HCC when administered directly after failure of ICIs. In that report, 36 cases in which ICIs were discontinued for ineffectiveness were then administered LEN. Consequently, the median OS following ICI therapy initiation was 29.8 months, which is about twice as long as the OS when LEN was started alone (16.7 months according to REFLECT).^[2]^ Thus, it is speculated that LEN after atezolizumab plus bevacizumab is considered to produce a good antitumor response.

There have been reports of conversion surgery for HCC patients performed after treatment with LEN.^[13,14]^ In our case, the adrenal metastasis did not shrink by atezolizumab plus bevacizumab followed by LEN, but the loss of contrast effect in the mRECIST evaluation led to the decision for conversion surgery. In histopathological examination, R0 resection was achieved, and there was almost no viable lesion at the site where the tumor had lost the contrast effect in the mRECIST evaluation. These results indicate that atezolizumab plus bevacizumab followed by LEN has a high degree of tumor necrosis effect, and that mRECIST is useful in determining the favorability of tumor resection. However, further case studies are required to verify this result.

## 4 Conclusion

In conclusion, the results from this case demonstrated that LEN after failure of atezolizumab plus bevacizumab was effective for HCC with adrenal metastasis and thus represents a bridge to successful surgery. Therefore, it is important to consider a variety of treatments given that there is a possibility of cure even when first-line treatment is not effective for a patient with unresectable HCC.

## Author contributions

**Supervision:** Tomokazu Kawaoka.

**Writing – original draft:** Shigeki Yano.

**Writing – review & editing:** Tomokazu Kawaoka, Yusuke Johira, Ryoichi Miura, Masanari Kosaka, Yuki Shirane, Serami Murakami, Kei Amioka, Kensuke Naruto, Yuwa Ando, Yumi Kosaka, Kenji Yamaoka, Kenichiro Kodama, Shinsuke Uchikawa, Hatsue Fujino, Atsushi Ohno, Takashi Nakahara, Eisuke Murakami, Wataru Okamoto, Masami Yamauchi, Michio Imamura, Keiichi Mori, Kouji Arihiro, Shintaro Kuroda, Tsuyoshi Kobayashi, Hideki Ohdan, Hiroshi Aikata.
